# IL‐1β Upregulates PD‐L1 in Small Extracellular Vesicle of Endothelial Origin

**DOI:** 10.1096/fj.202502508R

**Published:** 2025-11-23

**Authors:** Maria Cristina Gagliardi, Federica Felicetti, Lucia Bertuccini, Elena Ortona, Chiara Bolego, Katia Fecchi

**Affiliations:** ^1^ Department of Infectious Diseases Istituto Superiore di Sanità Rome Italy; ^2^ Department Oncology and Molecular Medicine IstitutoSuperiore di Sanità Rome Italy; ^3^ Core Facilities Technical Scientific Service IstitutoSuperiore di Sanità Rome Italy; ^4^ Center for Gender‐Specific Medicine Istituto Superiore di Sanità Rome Italy; ^5^ Department of Pharmaceutical and Pharmacological Sciences University of Padova Padova Italy

## Abstract

Programmed death ligand 1(PD‐L1) is of significant importance in the regulation of immune cells, and its levels increase in response to inflammation in HUVECs. In this letter, we examined the expression of PD‐L1 on small extracellular vesicles (sEVs) in human umbilical vein endothelial cells (HUVECs) and investigated its modulation in response to interleukin‐1β (IL‐1β), a key player in the immune response and is involved in the pathogenesis of several diseases. We found that stimulation of HUVECs with IL‐1β resulted in the release of a higher number of sEVs‐PD‐L1positive. This result provides significant insights into the potential role of sEVs‐PD‐L1positive in regulating inflammatory and immune responses in the vascular endothelium.

## Graphical abstract

PD‐L1 is crucial to the regulation of immune cells and rises in response to inflammation in HUVECs. We studied PD‐L1 expression on sEVs in HUVECs and explored its modulation in response to IL‐1β, a key player in the immune response and involved in the pathogenesis of several diseases. sEVs were collected by sequential ultracentrifugation from HUVEC (±IL‐β) and characterized by NTA, electron microscopy and WB. The workflow highlights that IL‐1β leads to the release of a greater quantity of sEVs‐PD‐L1^+^.

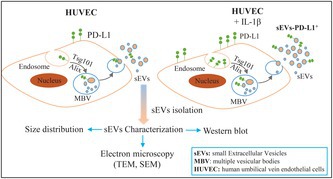

Programmed death protein‐1 (PD‐1) and its associated receptor programmed death ligand‐1 (PD‐L1) represent a significant regulatory axis of the immune system. PD‐1 is an inhibitory receptor of the CD28 family that is expressed on antigen‐activated T cells. PD‐L1 is expressed in a constitutive manner by a variety of hematopoietic cells, including T cells, dendritic cells and macrophages, as well as by endothelial cells (ECs) of the lungs, liver and intestine. PD‐1/PD‐L1 interaction negatively regulates the adaptive immune response by inhibiting the function of effector, especially CD8^+^ T cells, in peripheral tissues. Several diseases, including autoimmune diseases and cancer, have been linked to abnormal interactions between PD‐L1 and PD‐1. Checkpoint blockade with anti‐PD‐1 and anti‐PD‐L1 antibodies is used to treat a wide variety of cancers and is among the most efficient immunotherapies [1].

Evidence has been provided that the expression of PD‐L1 on ECs is crucial to the regulation of immune cell response by directly regulating lymphocyte activation and inhibiting the pathogenesis of several immunological diseases [2]. We previously showed that total PD‐L1 expression in human ECs, namely human umbilical vein endothelial cells (HUVECs), increases in response to inflammatory stimuli [3]. These cells are considered a valuable source for in vitro research on endothelial function. We also provided the first evidence that HUVECs from female but not male donors release a soluble form of PD‐L1 (sPD‐L1) in response to interleukin‐1β (IL‐1β) and Vascular Endothelial Growth Factor [3]. Increased levels of sPD‐L1 protein occur in a number of inflammatory pathologies possibly reflecting vascular inflammation; however, the biochemical function of sPD‐L1 remains to be elucidated [4, 5, 6]. In addition to being secreted as a circulating protein, PD‐L1 can be expressed on the surface of cell‐derived exosomes, which contribute to the circulating PD‐L1 pool [7]. Of note, often sPD‐L1 analysis is performed by immune‐assays (ELISA) that may not distinguish between exosome‐derived PD‐L1 and other soluble forms of PD‐L1.

Exosomes are small vesicles of 30–150 nm diameter surrounded by a lipid bilayer that are membrane‐bound and secreted by a variety of cell types. They are generated by the late endosomes of cells and secreted following the fusion of multiple vesicular bodies (MVBs) with the plasma membrane. MVBs are formed via a complex process subject to regulation by a variety of accessory proteins, including cluster of differentiation proteins (CD9, CD63, CD81), tumor susceptibility gene 101 protein (Tsg101) and programmed cell death 6‐interacting protein (Alix). This process involves either endosomal sorting complexes required for transport (ESCRT)‐dependent or ‐independent mechanisms. Secreted exosomes are involved in several cellular processes including paracrine and autocrine cell–cell communication due to their ability to carry functional molecules (proteins, messenger RNAs, small non‐coding microRNAs, mitochondrial DNA and lipids) from originating to target cells [8].

To date, no specific markers of EV subtypes have been identified that allow their endosomal or plasma membrane origin to be determined [9]. As assigning an EV population to a specific biogenesis pathway remains challenging, we will refer to secreted vesicles using the term “small extracellular vesicles” (sEVs) as recommended by the 2023 Minimal Information for Studies of Extracellular Vesicles (MISEV) guidelines [10]. Furthermore, it has been shown that PD‐L1 expression is enriched on the surface of sEVs derived from tumor cells [11].

Thus, we set out to assess PD‐L1 expression on sEVs (sEVs‐PD‐L1^+^) in HUVECs and to explore its modulation in response to IL‐1β, which is a major player in the immune response and is involved in the pathogenesis of several diseases including cancer [12].

Experiments were conducted using HUVECs from female donors isolated from human umbilical cords and cultured as previously described [13]. HUVECs were stimulated with IL‐1β (0.5 ng/mL) in a medium with exosome‐depleted FCS for 24 h. Conditioned medium was then harvested, subjected to sequential ultracentrifugation and characterized as illustrated in Figure 1.

**FIGURE 1:**
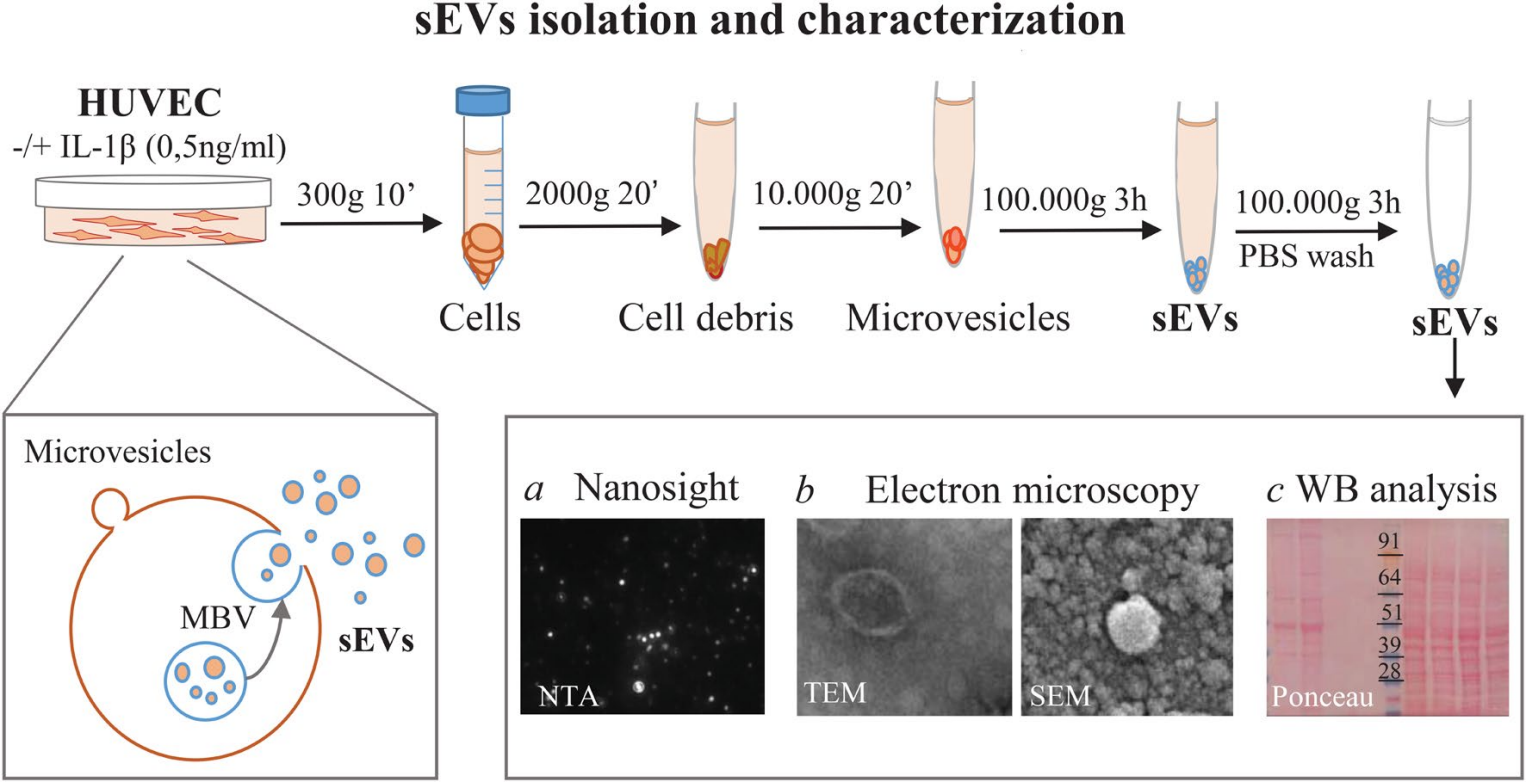
Schematic drawing for small Extracellular Vesicles (sEVs) isolation procedure by differential ultracentrifugation. HUVEC cells were seeded in dishes and treated with IL‐1β (0.5 ng/mL) in medium with exosome‐depleted FCS for 24 h. At the end of treatment, culture supernatants were harvested at 4°C and clarified of cellular debris by spinning at 2000 g for 20 min at 4°C. The culture supernatants were then ultracentrifuged with a SW41Ti rotor at 10 000 *g* for 20 min to eliminate macrovesicles and various aggregates. sEVs containing exosomes were isolated by ultracentrifugation at 100 000 *g* for 3 h. The pellet was then washed with PBS at 100 000 *g* for 3 h. sEVs collected were physically and biochemically characterized by (a) Nanoparticle Tracking Analysis (NTA) to evaluate particle size distribution and concentration. (b) Transmission and scanning electron microscopy (TEM and SEM, respectively) to identify morphology. (c) Western blot analysis to detect sEVs surface markers. Insets are illustrative representations images of sEVs from purified by HUVEC.

We first investigated the morphology of sEVs secreted by HUVECs using scanning and transmission electron microscopy (SEM and TEM, respectively). SEM analysis shows a general view of the different sEV populations purified from control and IL‐1β‐treated cell monolayers, and reveals a higher density of sEVs in the latter. Furthermore, TEM analysis shows that isolated vesicles were membrane‐bound in both samples (Figure 2A). The size distribution and concentration of sEVs were determined by nanoparticle tracking analysis (NTA). According to NTA measurements, vesicles obtained from HUVECs treated or not with IL‐1β were found to be homogeneous in size, with a mode of 120–150 nm, the size range commonly attributed to sEVs. IL‐1β‐treated cells produced a significantly higher concentration of sEVs compared to untreated cells as determined by quantitative analysis (Figure 2B). The exosomal identity of collected sEVs was confirmed by Western blotting for TSG101, Alix and CD81 expression, which are commonly used as marker proteins for sEVs. Interestingly, PD‐L1 expression levels increased remarkably following IL‐1β treatment (Figure 2C). In conclusion, the present results show for the first time that stimulation of HUVECs with IL‐1β results in the release of a higher number of sEVs‐PD‐L1^+^.

**FIGURE 2:**
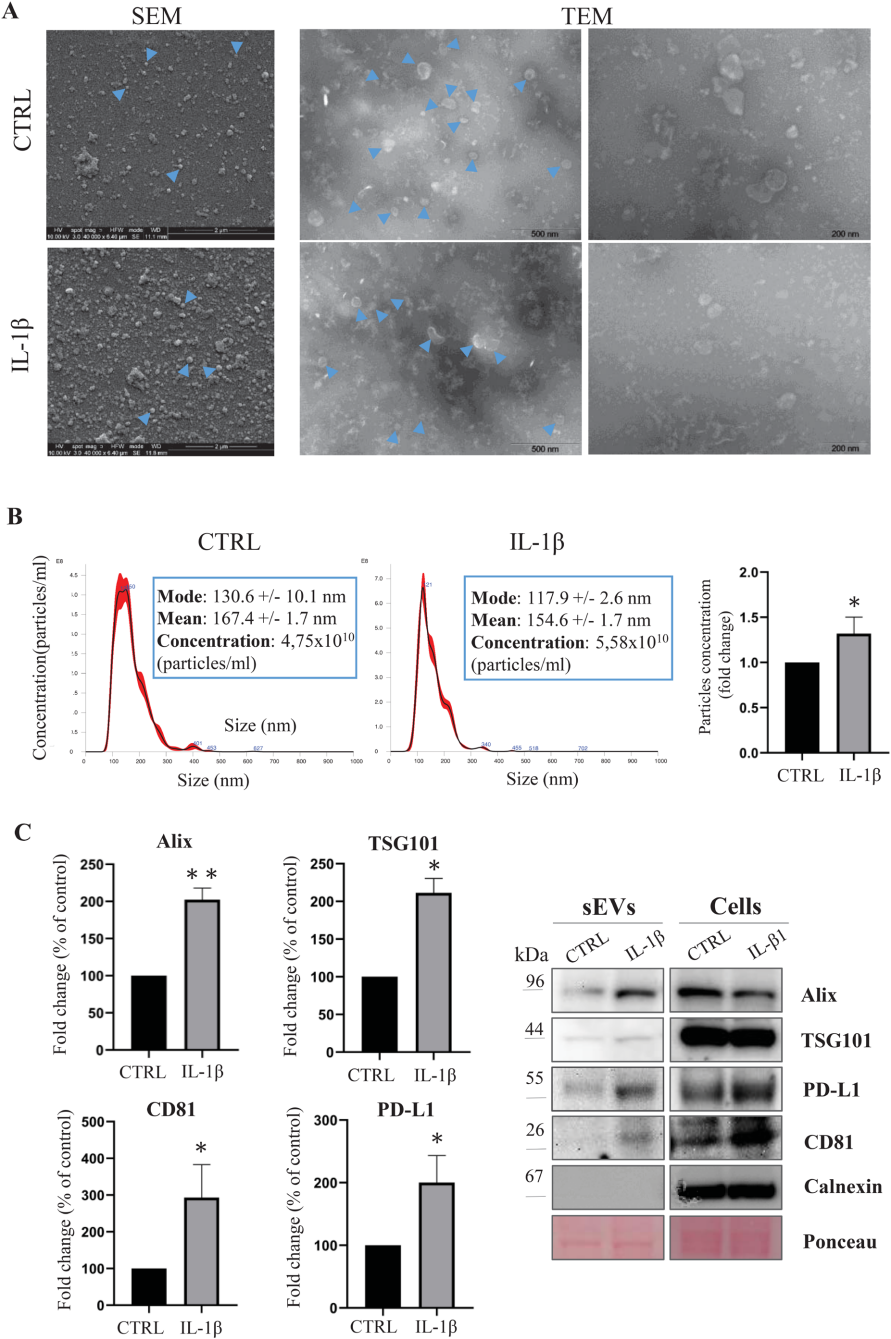
Characterization of sEVs derived from HUVECs. (A) Electron microscopy analysis of sEVs purified from HUVEC (± IL‐1β). SEM (left panel) and TEM (right panel) analysis of sEVs purified from cell supernatants. SEM micrographs show an overview of the different vesicle populations purified from control (upper panel) and IL‐1β‐treated (lower panel) monolayers. Scale bar, 2 μm. TEM micrographs illustrate the membranous nature and round shape morphology of isolated vesicles in both control and IL‐β groups. Images are representative of three independent experiments. Scale bars, 500 nm (left panel) and 200 nm (right panel). Arrows exemplarily highlight vesicular structures. (B) NTA analyses of sEVs isolated by sequential ultracentrifugation from supernatants of HUVEC cells (±IL‐1β). A representative curve showing vesicle size distribution (nm) and concentration (particles/mL) is shown. Each curve was generated from three measurements per sample and an average value was given. The mean, mode, and concentration are reported adjacent to the curve. The histogram illustrates the concentration of sEVs samples as fold change of control, according to NTA measures (*n* = 5 independent experiments, *p < 0.05). The data were evaluated statistically by unpaired *t*‐test using GraphPad Prism Software, version 8.0.2. (C) Biochemical characterization of sEVs from HUVECs. sEVs collected by sequential ultracentrifugation from HUVEC (±IL‐1β) were resuspended with the same volume for each condition and loaded for Western blot analysis. Representative images show exosome markers (Alix, TSG101, CD81) and PD‐L1 detection. In the right panel, representative images of the same protein pattern obtained from cells is shown for comparation. Ponceau red was used as a loading control. Protein levels were quantified by densitometric analysis. Data are expressed as percent versus control (CTRL) and represent the mean ± SEM of 3 independent experiments. Statistical analysis was performed using using GraphPad Prism Software, version 8.0.2. Unpaired *t*‐test (*p < 0.05, **p < 0.01).

Recent studies have revealed that sEVs‐PD‐L1^+^ derived from tumors are able to suppress antitumor immunity locally and systemically through ligation of PD‐1 on T cells by suppressing their proliferation, survival and effector function and thus facilitating immune escape and tumor progression [14]. Moreover, it has also been shown that sEVs‐PD‐L1^+^ derived from tumor cells promote tumor growth in vivo, including cancers of the breast and prostate, colorectal cancer, melanoma, and non‐small cell lung cancer [15, 16]. Higher levels of sEVs‐PD‐L1^+^ have been associated with a poor prognosis in a variety of tumor types [17] suggesting that sEVs‐PD‐L1^+^ are a promising biomarker for diagnosing and prognosing cancer, as well as for predicting responses to immune therapies. Although frequently measured in plasma, sEVs‐PD‐L1^+^ can also be detected in other body fluids, such as saliva, urine, or cerebrospinal fluid. For example the presence of sEVs‐PD‐L1^+^ in urine samples from patients with urothelial cancer is associated with responses to anti‐PD‐L1 therapy [18]. However, the expression of sEVs‐PD‐L1^+^ does not always have a negative impact. Its functions beyond cancer have recently been highlighted. Excessive and persistent inflammation after injury can result in chronic wounds and tissue damage. Inhibiting overactive immune cells at the wound site can promote effective repair. Su et al. showed that sEVs‐PD‐L1^+^ could promote epidermal cell and dermal fibroblast migration, suggesting a role for exoPD‐L1 in wound healing [18].

Overall, the potential of sEVs‐PD‐L1^+^ as an immunosuppressive agent is promising for a wide range of therapeutic applications, including the treatment of disorders such as autoimmune diseases, chronic infections, and inflammation [19].

Further research will clarify the mechanisms involved in the release of sEVs‐PD‐L1^+^ subsequent to inflammatory stimuli in the vascular endothelium and in other cell types such as macrophages and cancer cells. This will establish the basis for elucidating the potential role of sEVs‐PD‐L1^+^ in modulating the inflammatory and immune response under physiological and pathological conditions.

## Author Contributions

**Maria Cristina Gagliardi:** designed the work, performed the experiments, analyzed the results. **Federica Felicetti:** acquired and analyzed nanoparticle tracking analysis. **Lucia Bertuccini:** acquired and analyzed electron microscopy data. **Chiara Bolego and Elena Ortona:** critically revised the manuscript. **Katia Fecchi:** contributed to conception and design all the experiments, supervised data acquisition and analysis and wrote the manuscript.

## Funding

The authors have nothing to report.

## Conflicts of Interest

The authors declare no conflicts of interest.

## Data Availability

Data supporting this study included in the article.
